# Anthocyanins: From Mechanisms of Regulation in Plants to Health Benefits in Foods

**DOI:** 10.3389/fpls.2021.748049

**Published:** 2021-10-28

**Authors:** Francesca Cappellini, Alessandra Marinelli, Marta Toccaceli, Chiara Tonelli, Katia Petroni

**Affiliations:** Dipartimento di Bioscienze, Università degli Studi di Milano, Milano, Italy

**Keywords:** MYB, bHLH, WD40, transcription factors, anthocyanins, chronic diseases

## Abstract

Anthocyanins represent the major red, purple, and blue pigments in many flowers, fruits, vegetables, and cereals. They are also recognized as important health-promoting components in the human diet with protective effects against many chronic diseases, including cardiovascular diseases, obesity, and cancer. Anthocyanin biosynthesis has been studied extensively, and both biosynthetic and key regulatory genes have been isolated in many plant species. Here, we will provide an overview of recent progress in understanding the anthocyanin biosynthetic pathway in plants, focusing on the transcription factors controlling activation or repression of anthocyanin accumulation in cereals and fruits of different plant species, with special emphasis on the differences in molecular mechanisms between monocot and dicot plants. Recently, new insight into the transcriptional regulation of the anthocyanin biosynthesis, including positive and negative feedback control as well as epigenetic and post-translational regulation of MYB-bHLH-WD40 complexes, has been gained. We will consider how knowledge of regulatory mechanisms has helped to produce anthocyanin-enriched foods through conventional breeding and metabolic engineering. Additionally, we will briefly discuss the biological activities of anthocyanins as components of the human diet and recent findings demonstrating the important health benefits of anthocyanin-rich foods against chronic diseases.

## Introduction

Anthocyanin synthesis is one of the most studied biosynthetic pathways in plants. Besides providing the major red, purple, violet, and blue pigmentation in flowers and fruits for attracting pollinators and seed dispersers, anthocyanins act as antioxidants in plants and are involved in both abiotic and biotic stresses, such as UV radiation, cold temperatures, drought, and in defense against pathogens and herbivores (Sarma and Sharma, 1999; Lorenc-Kukuła et al., 2005; Gould and Lister, 2006). Anthocyanins are health-protecting components of our daily diet found to activate endogenous antioxidant defenses and suppress inflammatory mediators (Speciale et al., 2013; Lee et al., 2014; Ullah et al., 2019; Kozłowska and Dzierżanowski, 2021). A number of studies suggest that they have protective effects against cardiovascular diseases, obesity, cancer, and neurodegenerative diseases (Tsuda, 2012; Li et al., 2017b; Salehi et al., 2020). For this reason, there has been growing interest in the identification of regulatory genes controlling anthocyanin biosynthesis in major crops as targets for both metabolic engineering and breeding programs.

**Figure 1:**
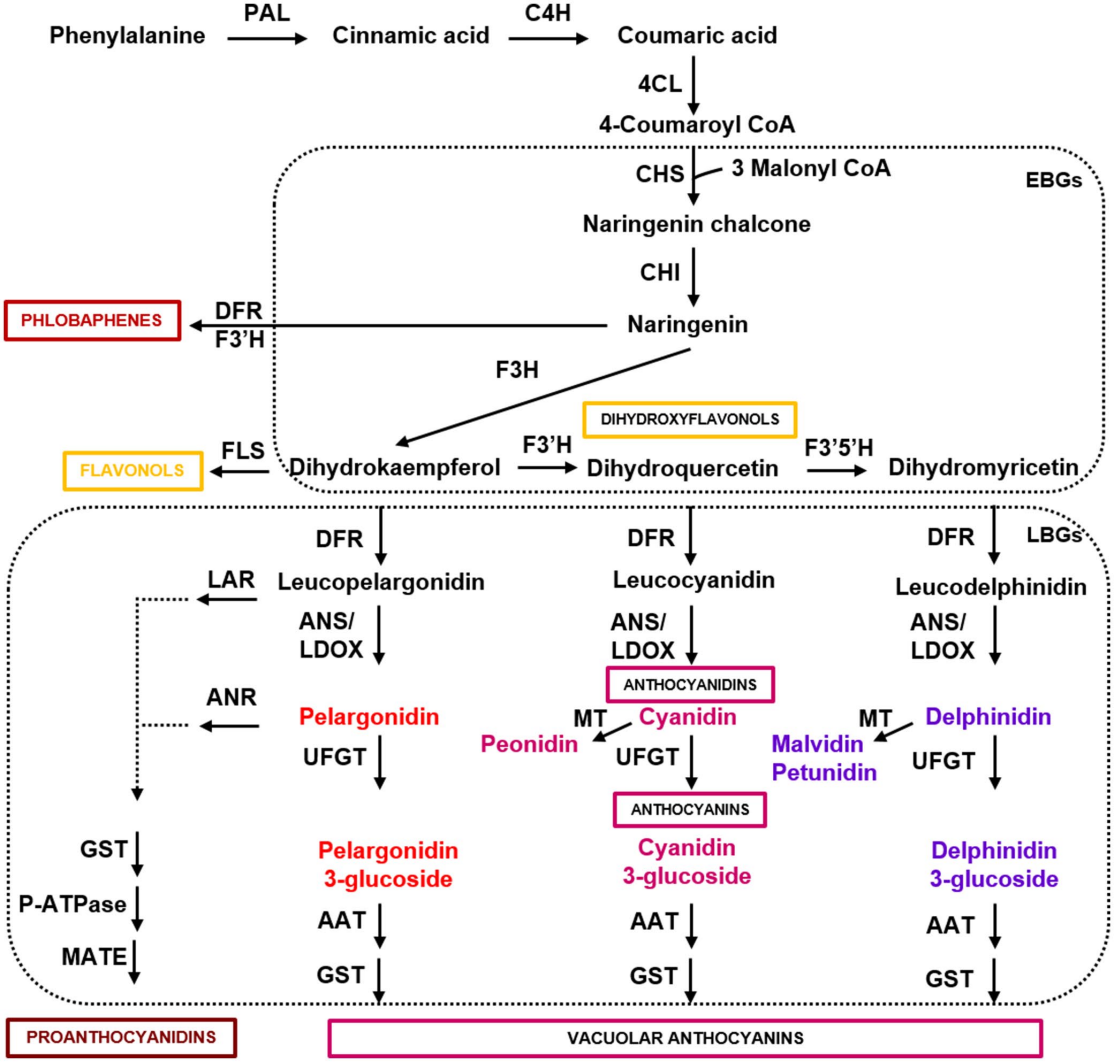
Simplified scheme of the flavonoid pathway, comprising the general phenylpropanoid pathway, the anthocyanin branch, and other subgroups of flavonoid end-products. Enzyme names are abbreviated as follows: PAL, phenylalanine ammonia lyase; C4H, cinnamic acid 4-hydroxylase; 4CL, 4 coumarate CoA ligase; CHS, chalcone synthase; CHI, chalcone isomerase; F3H, flavanone 3-hydroxylase; F3′H, flavanone 3′-hydroxylase; F3′5′H, flavanone 3′5′-hydroxylase; DFR, dihydroflavonol reductase; FLS, flavonol synthase; ANS/LDOX, anthocyanidin synthase/leucoanthocyanidin dioxygenase; UFGT, UDP-flavonoid glucosyl transferase; ANR, anthocyanidin reductase; LAR, anthocyanidin reductase; MT, methyltransferase; AAT, anthocyanin acyltransferase; GST, glutathione S-transferase; P-ATPase, P-type ATPase proton pump; MATE, MATE-type transporter. Boxes indicate the early and late biosynthetic genes (EBGs and LBGs) of the flavonoid pathway in dicots.

Anthocyanins are the final products of a specific branch of flavonoid biosynthesis, also producing flavonols, phlobaphenes, and proanthocyanidins (Figure 1). Flavonols, proanthocyanidins, and anthocyanins are water-soluble and almost ubiquitous, whereas phlobaphenes are alcohol-soluble and water-insoluble phenolics mostly produced in maize, wheat, and sorghum (Casas et al., 2014; Ibraheem et al., 2015; Lachman et al., 2017). The first biosynthetic genes of the anthocyanin pathway (i.e., *CHS*, *chalcone synthase*; *CHI*, *chalcone isomerase*; *F3H*, *flavanone 3-hydroxylase*; *F3′H*, *flavanone 3′-hydroxylase*; *F3′5′H*, *flavanone 3′5′-hydroxylase*), which have been termed early biosynthetic genes (EBGs), are involved in the synthesis of dihydroflavonols (i.e., dihydrokaempferol, dihydroquercetin, and dihydromyricetin). Dihydroflavonols are then converted into anthocyanidins (i.e., pelargonidin, cyanidin, and delphinidin) by the late biosynthetic genes (LBGs), encoding dihydroflavonol reductase (DFR), flavonol synthase (FLS), and anthocyanidin synthase/leucoanthocyanidin dioxygenase (ANS/LDOX), and to the anthocyanidin derivatives peonidin, malvidin, and petunidin by methyltransferases (MT; Figure 1). Anthocyanidins are glycosylated by UDP-glucose flavonoid 3-O-glucosyltransferase (UFGT), acylated by anthocyanin acyltransferase (AAT), and then transferred to the vacuole by glutathione S-transferase (GST). The stability of different anthocyanins is highly influenced by glycosylation and acylation, both aromatic and aliphatic, and influence their color and degradation in plant tissues (Glover and Martin, 2012). Anthocyanin 3-monosaccharides (e.g., 3-glucoside) are the most common, anthocyanin 3-disaccharides (e.g., 3-rutinoside) are generally more stable than 3-monosaccharides, whereas anthocyanin 3,5 disaccharides are also common, but less stable (Zhang et al., 2014a). The sugar residues of anthocyanins are often acylated with aromatic (e.g., p-coumaric, caffeic, and ferulic) or aliphatic acids (e.g., malonic and acetic acid). Anthocyanin acylation increases the stability, allowing intra- and inter-molecular co-pigmentation, and causes a shift toward the blue color (Zhang et al., 2014a).

Anthocyanin biosynthesis has been studied extensively, and key regulatory genes have been identified in many plant species (Petroni and Tonelli, 2011; Patra et al., 2013c; Chaves-Silva et al., 2018; Liu et al., 2018). Here, we focus on recent progress in understanding the positive and negative regulatory mechanisms of anthocyanin biosynthesis. Activators and repressors of anthocyanins in maize and Arabidopsis will be firstly described as examples of the regulatory systems in monocots and dicots, respectively, with particular emphasis on the important post-transcriptional mechanisms of regulation involving miRNAs identified in Arabidopsis and thereafter in other dicots. Finally, recent findings demonstrating the important health benefits of anthocyanin-rich food against chronic diseases will be discussed.

## Activators of Anthocyanin Biosynthesis in Maize and Arabidopsis

**Figure 2:**
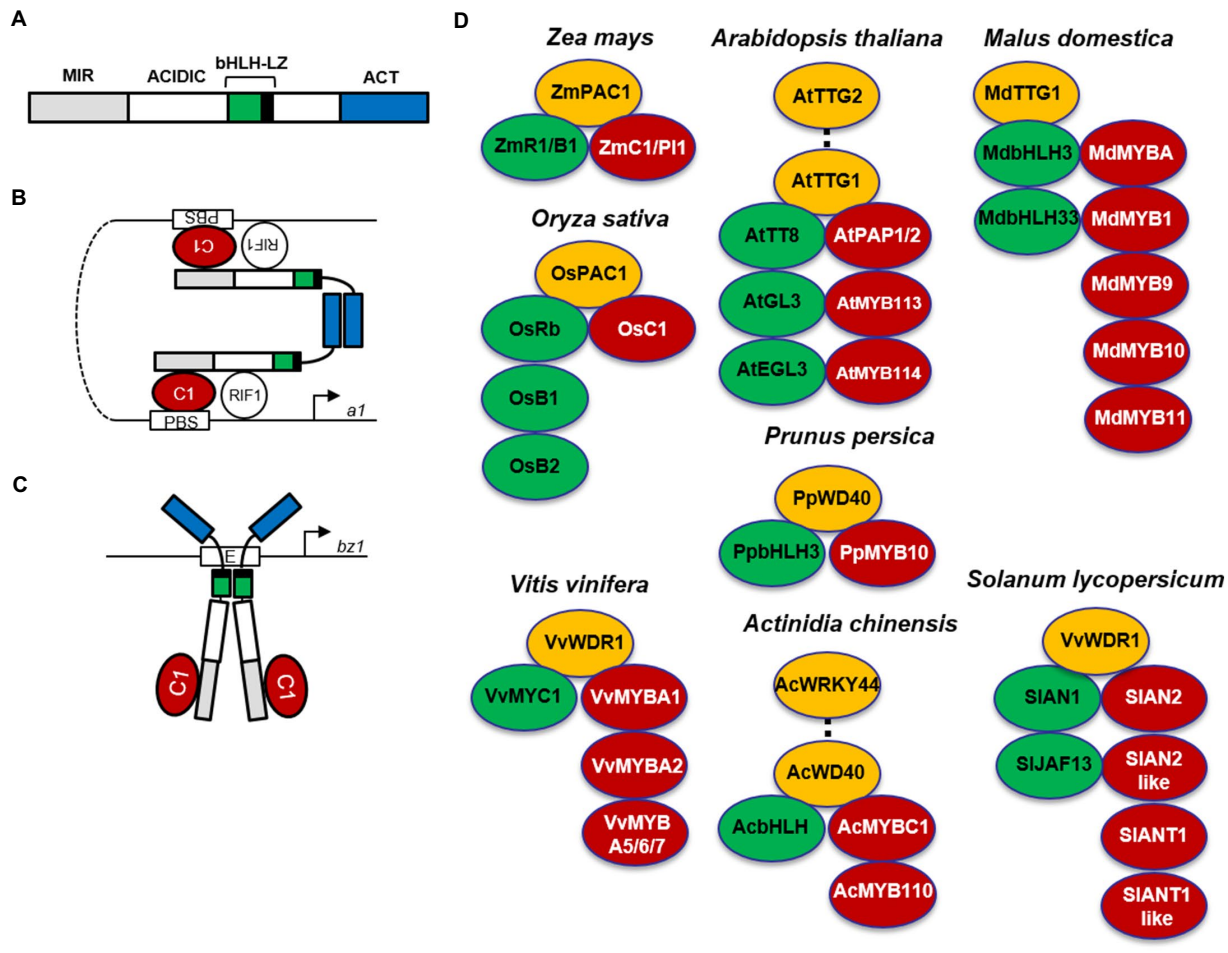
MBW complexes in different plant species. Alternative R1/C1 complexes activating anthocyanin biosynthesis genes in maize. **(A)** Schematic representation of the R1 functional domains. MIR, MYB interacting region; ACIDIC, acidic region; bHLH, basic helix-loop-helix domain; LZ, leucine zipper; ACT, ACT-like domain. **(B)** In the first R1/C1 complex, R1 homodimerizes *via* the ACT-like domain, the bHLH region of R1 interacts with R-interacting factor 1 (RIF1), and the N-terminal acidic region interacts with C1, which binds the P1-binding sites (PBS) found in promoters of some anthocyanin biosynthesis genes, such as a1. **(C)** In the second R1/C1 complex, R1 homodimerizes *via* the bHLH-LZ domain, which binds E-box elements, such as those present in the bz1 promoter, and interacts with the C1 protein, providing a strong transcriptional activation domain. (Schemes modified from Kong et al., 2012). **(D)** MYB-bHLH-WD40 (MBW) complexes involved in anthocyanin biosynthesis in different plant species. MYBs are indicated in red, bHLHs in green, and WD40s in yellow. The Arabidopsis MBWW complex (PAP1-TT8-TTG1-TTG2) controlling a subset of proanthocyanidin LBGs is indicated by a dotted line connecting TTG2 and TTG1. The MBWW complex (AcMYBC1-AcbHLH-AcWD40-AcWRKY44) controlling *F3′H* and *F3′5′H* in kiwifruit (*Actinidia chinensis*) is indicated by a dotted line connecting AcWD40-AcWRKY44.

In most species, anthocyanin biosynthesis is activated by the interaction of R2R3-MYB regulatory proteins in combination with bHLH and WD40-type regulatory proteins to form a ternary MBW complex (Figure 2; Allan et al., 2008; Petroni and Tonelli, 2011; Chaves-Silva et al., 2018).

In the monocot maize, the regulatory genes of anthocyanin biosynthesis are divided in two families (*C1/Pl1* and *R1/B1*), encoding MYB and bHLH transcription factors, respectively. The *R1/B1* genes (*R1*, *Red1*; *B1*, *Booster1*; *Sn1*, *Scutellar node1*; *Lc1*, *Leaf color1*; *Hopi1*), whose expression is tissue-specific, determine the distribution of pigment production in different tissues, whereas *Colorless1* (*C1*) in the seed or *Purple plant1* (*Pl1*) in plant tissues regulates anthocyanin accumulation during development and in response to light. In maize seed, the dominant *R1 C1* genes activate anthocyanin pigmentation of the aleurone layer in blue corn, whereas the dominant *B1 Pl1* genes induce anthocyanin synthesis in the pericarp of purple corn (Petroni et al., 2014). MYB and bHLH transcription factors form a heterodimer activating all anthocyanin biosynthetic genes in a coordinate manner (Hernandez et al., 2004). By contrast, the phlobaphene branch only requires the maize MYB PERICARP COLOR1 (P1) to activate a subset of biosynthetic genes (i.e., *CHS*, *CHI*, *DFR*, and *FLS*; Ferreyra et al., 2010). In addition, the PALE ALEURONE COLOR1 (PAC1) WD40 protein cooperates with B1 or R1 proteins for full activation of anthocyanin biosynthetic genes in the seed, probably *via* a ternary MBW complex similar to that identified in other species (Figure 2D; Carey et al., 2004).

Despite being activated coordinately, anthocyanin biosynthetic genes lack conserved cis-regulatory elements. Therefore, their coordinate activation is proposed to be achieved by two different R1/C1 complexes, each able to bind specific cis-elements and thus to activate a subset of anthocyanin biosynthetic genes (Tuerck and Fromm, 1994). These alternative R1/C1 complexes depend on two different dimerization domains found in bHLH proteins (Figure 2A). In the proposed model, when R1 homodimerizes *via* the ACT-like domain (Figure 2B), the bHLH region of R1 interacts with R-interacting factor 1 (RIF1), an EMSY-like maize nuclear factor, necessary to the R1/C1 complex for transcriptional activation of anthocyanin biosynthetic genes through increased histone acetylation of the promoter region (Hernandez et al., 2007), whereas the N-terminal acidic region interacts with the MYB C1, necessary to bind the P1-binding sites (PBS) in the a1 promoter (Sainz et al., 1997). When R1 homodimerizes *via* the bHLH-LZ domain (Figure 2C), it directly binds E-box elements in the *bronze1* (*bz1*) promoter, and interacts with the C1 protein, necessary to provide a strong transcriptional activation domain (Kong et al., 2012).

In the dicot Arabidopsis, EBGs leading to the production of flavonols (Figure 1) are activated by functionally redundant *R2R3-MYB* regulatory genes (*MYB11/12/111*), whereas the activation of LBGs, leading to the production of proanthocyanidins in seeds and anthocyanins in vegetative tissues (Figure 1), requires the MYB-bHLH-WD40 complex (Figure 2D; Baudry et al., 2004; Stracke et al., 2007; Gonzalez et al., 2008). The MBW complex activating anthocyanin synthesis in vegetative tissues includes the WD40 factor TRANSPARENT TESTA GLABRA1 (TTG1) and different bHLH and MYB transcription factors (Figure 2D). The *bHLH TRANSPARENT TESTA8 (TT8)*, *GLABRA3 (GL3)*, and *ENHANCER OF GLABRA3 (EGL3)* genes have partially redundant roles in the regulation of anthocyanins in seedlings (Zhang et al., 2003; Gonzalez et al., 2008). Among R2R3-MYB factors, MYB75/PRODUCTION OF ANTHOCYANIN PIGMENT1 (PAP1), MYB90/PRODUCTION OF ANTHOCYANIN PIGMENT2 (PAP2), MYB113, and MYB114 can form multiple MBW complexes with EGL3, GL3, and TT8 (Gonzalez et al., 2008). On the other hand, seed-specific activation of proanthocyanidin synthesis requires the activity of an MBW complex comprising the R2R3-MYB protein MYB123/TRANSPARENT TESTA2 (TT2), TT8, and TTG1 (Ramsay and Glover, 2005). A newly identified complex requires the additional interaction between TTG1 and TTG2 WRKY transcription factors, thus forming an MBWW complex necessary to activate a subset of LBGs (i.e., *TRANSPARENT TESTA12*, *TT12* and *Autoinhibited H(+)-ATPase Isoform 10*, *AHA10*) encoding a MATE type transporter and a P-type ATPase proton pump, functioning as vacuolar proanthocyanidin transporters (Figure 2D; Gonzalez et al., 2016; Lloyd et al., 2017). When overexpressed, *PAP1* and *PAP2* also weakly activate EBGs (Tohge et al., 2005), but in *pap1* knock-out plants or when all four MYBs (*PAP1*, *PAP2*, *MYB113*, and *MYB114*) were silenced through RNAi, only LBGs were found to be significantly reduced (Gonzalez et al., 2008).

Interestingly, a two-step activation of anthocyanin biosynthesis has been proposed in Arabidopsis (Albert et al., 2014). TT8 is regulated at the transcriptional level and can regulate its own expression through a positive feedback mechanism. In etiolated seedlings, an initial PAP1/EGL3/TTG1 complex may activate *TT8*, which can then form MBW complexes that autoactivates *TT8* through a positive feedback mechanism and confers a strong induction of LBGs and anthocyanin accumulation (Baudry et al., 2006). In seedlings, *TT8* expression is therefore controlled by three different MBW complexes, including PAP1, TTG1, and one of three bHLHs (TT8/EGL3/GL3), whereas three other MBW complexes consisting of TTG1, TT2/MYB5, and TT8/EGL3 control proanthocyanidin accumulation in seeds (Xu et al., 2015). Overall, it appears that despite the variable function of transcription factors in the MBW complex, the complex is conserved in monocots and dicots, but the mechanisms of regulation of the regulatory genes have diverged. In the monocot maize, each regulatory gene is independently regulated, whereas in dicots a regulatory loop exists, in which one regulatory gene controls the expression of another in a tissue- and developmental-specific manner (Carey et al., 2004; Xu et al., 2015). Consistent with this divergence, based on phylogenetic analyses, the R2R3-MYBs from Arabidopsis (i.e., MYB75/PAP1, MYB90/PAP2, MYB113, and MYB114) and many other angiosperms belong to subgroup 6, whereas the R2R3-MYBs C1 and Pl1 from maize are more similar to R2R3-MYBs from subgroup 5 (i.e., TT2), which usually activate proanthocyanidins (Dubos et al., 2010).

Considerable research on the upstream activators of MBW complexes that mediate the response to environmental signals has been reported in many reviews (Jaakola, 2013; Zoratti et al., 2014; Xu et al., 2015; Chaves-Silva et al., 2018; Liu et al., 2018), but only ELONGATED HYPOCOTYL 5 (HY5) has been demonstrated as a direct activator of *PAP1* in response to light through direct binding to the G-box and ACE element in the *PAP1* promoter (Shin et al., 2013). Therefore, HY5 appears to regulate anthocyanin biosynthesis in two ways: *via* direct binding to the promoter regions of the biosynthetic genes, and by positive regulation of *PAP1* transcription (Shin et al., 2013). Other candidates in the light-regulated activation of *PAP1* are LIGHT-REGULATED ZINC FINGER PROTEIN 1 (LZF1), which is under control of HY5 (Chang et al., 2008), the NAC domain transcription factor, ANAC078, that also activates *TT2*, *TT8*, *GL3*, and *EGL3* in addition to *PAP1* (Morishita et al., 2009), and MYB112 which activates the expression of *PAP1* upon perception of high light and salt stress, but acts negatively toward *MYB12* and *MYB111*, which both control flavonol biosynthesis (Lotkowska et al., 2015).

**Figure 3:**
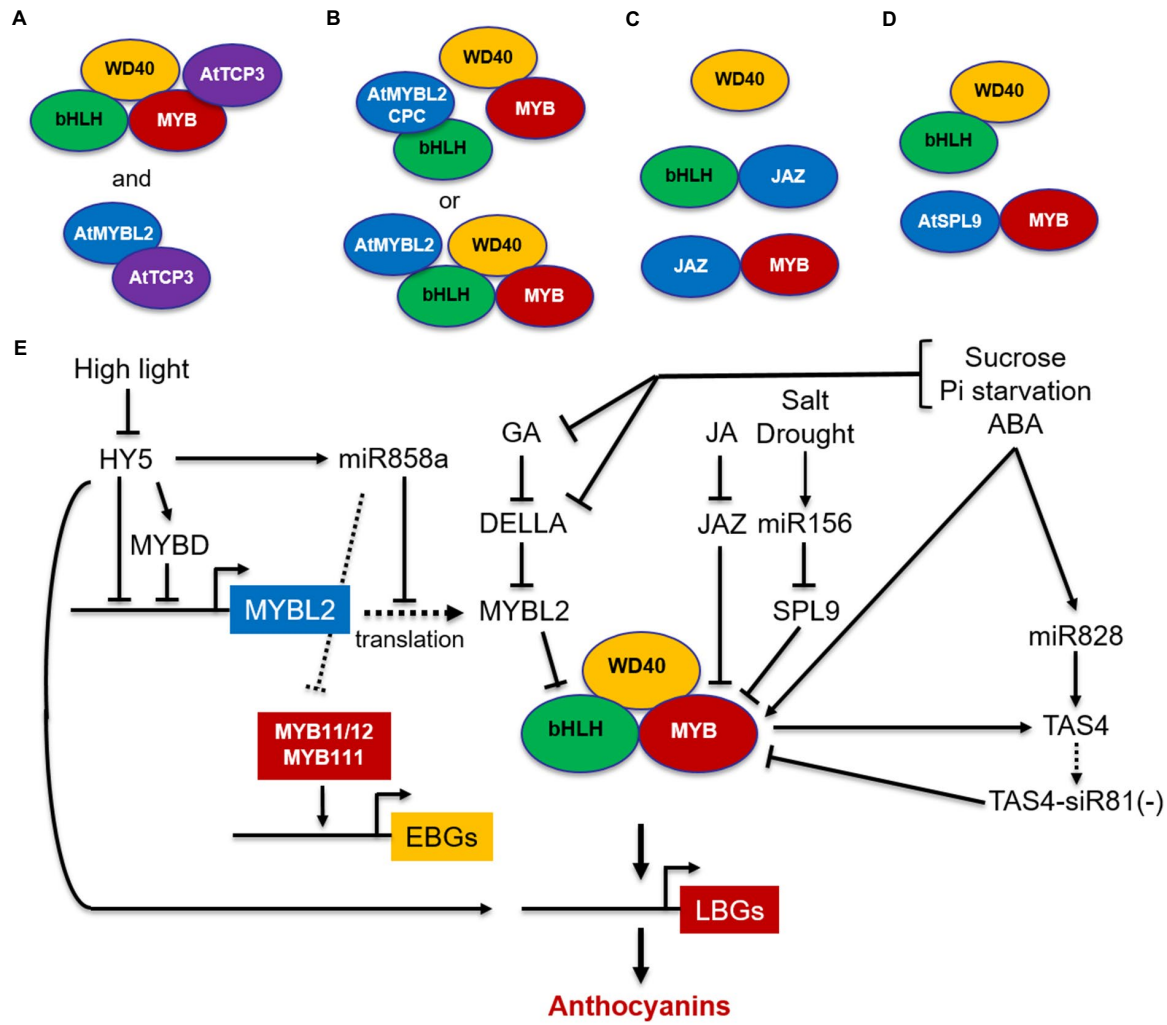
Schematic model of positive and negative regulation of MBW complexes in Arabidopsis. The MYB-bHLH-WD40 (MBW) complex activates the expression of LBGs. **(A)** TCP3 stabilizes the MBW complex and reinforces its transcriptional capacity by associating with MYBs and by interacting with the MYBL2 repressor, thus preventing its binding to the bHLH proteins; **(B)** MYBL2 disrupts the MBW complex by interacting with bHLHs or participates in the MBW complex and down-regulates LBGs through its repression domain; **(C)** JAZ proteins disrupt the MBW complex by interacting with bHLHs or R2R3-MYBs; **(D)** SPL9 disrupts the MBW complex by interacting with MYBs; **(E)** simplified model for negative regulation of *MYB11/12/111*genes and the MBW complex through MYBL2, JAZ, SPL9, and miRNAs. MYBs are indicated in red, bHLHs in green, and WD40s in yellow. TCP3 activator is indicated in violet, whereas MYBL2 and SPL9 repressors are indicated in light blue. Arrows, positive regulation; blunt ends, negative regulation; dashed arrow, translation.

Interestingly, the MBW complexes are then stabilized by the transcription factor TCP3, which interacts with R2R3-MYBs and reinforces their transcriptional activation capacity when bound to TT8, thus promoting the expression of LBGs (Figure 3A). TCP3 also interacts with R2R3-MYBs controlling EBGs (MYB11/MYB12/MYB111), thereby enhancing both flavonol and anthocyanin production (Li and Zachgo, 2013). In yeast 2-hybrid, TCP3 was also found to form a heterodimer with the negative regulator R3-MYB protein MYB-LIKE2 (MYBL2; see below), suggesting that TCP3 may stabilize the MBW complex by sequestering MYBL2 and preventing its binding to the bHLH proteins (Figure 3A; Li and Zachgo, 2013). Therefore, TCP3 can be considered an anthocyanin activator with a positive activation mode, based on the interaction with the MBW complex to enhance its transcriptional activity, and a passive activation mode, based on the sequestration of the MYBL2 repressor, known to fine-tune anthocyanin accumulation (Figure 3A).

## Repression of Anthocyanin Biosynthesis in Maize and Arabidopsis

Significant progress in understanding the negative regulators controlling flavonoid biosynthesis has been made (Li, 2014; Xu et al., 2015; Chen et al., 2019; LaFountain and Yuan, 2021). Most anthocyanin repressors act by disrupting the MBW complexes, but there is increasing evidence of direct repression of biosynthetic genes, post-transcriptional regulation through miRNAs (see also Section “Post-transcriptional Regulation of Anthocyanin Biosynthesis in Arabidopsis and Other Dicots”) and post-translational modifications that fine-tune the activity of MBW complexes.

In maize, the *INTENSIFIER1* (*IN1*) gene encodes a bHLH protein, which is most closely related to the clade bHLH-2 within subgroup IIIf of the bHLH family (Feller et al., 2011). The bHLH-1 proteins regulate anthocyanin biosynthesis, whereas in many plants the bHLH-2 proteins, besides being essential for anthocyanin biosynthesis (Albert et al., 2021), are also involved in regulating proanthocyanidin biosynthesis and vacuolar acidification (Spelt et al., 2002; Butelli et al., 2019; Strazzer et al., 2019). In maize, the *IN1* gene inhibits the anthocyanin pathway, possibly by interfering with R1 binding in the MBW complex, since IN1 does not appear to control the transcript level of the *B1* and *C1* genes (Carey et al., 2004; Feller et al., 2011).

In dicots, the first negative *R2R3-MYB* regulators identified, *AmMYB308* and *AmMYB330* from *Antirrhinum majus*, reduced anthocyanin biosynthesis when overexpressed in tobacco flowers (Tamagnone et al., 1998), whereas in Arabidopsis *AtMYB4* was the first *R2R3-MYB* shown to act as a repressor of phenylpropanoid metabolism (Jin et al., 2000). Both contain an *ETHYLENE RESPONSE FACTOR* (ERF)-associated amphiphilic repression (EAR) motif in the C-terminal domain and belong to subgroup 4, where many other plant R2R3-MYB repressors are included (Dubos et al., 2010; Chen et al., 2019).

In addition to R2R3-MYB repressors, in Arabidopsis two types of R3-MYB repressors, MYBL2 and CAPRICE (CPC), have been identified. MYBL2 is an R3-MYB repressor derived from subgroup 4 R2R3-MYBs after a partial loss of the R2 domain and characterized by an R3 domain including the bHLH interacting motif and a C-terminal domain containing an EAR motif and a TLLLFR repression motif (Dubos et al., 2008; Matsui et al., 2008). Conversely, CPC consists of a short protein, containing the highly conserved bHLH-interacting motif, but no repressor domains, and belongs to a distinct clade that emerged before the divergence between monocots and dicots (Albert et al., 2014). Specifically, CPC is known to regulate root hair differentiation, trichome initiation, and stomatal formation (Wada et al., 1997), but it has also been shown to be involved in the regulation of anthocyanin biosynthesis in Arabidopsis (Zhu et al., 2009). These two types of R3-MYB repressors are characterized by two different mechanisms of action: CPC only exerts passive repression by titrating the bHLH partners from the MBW complex (Zhu et al., 2009), whereas MYBL2 exerts both a passive repression by preventing the formation of an MBW complex through interaction with the bHLH proteins and an active repression by participating in the MBW complex and actively repressing transcription of LBGs *via* an EAR repression domain (Figure 3B; Dubos et al., 2008; Matsui et al., 2008). In addition, MYBL2 represses the expression level of *MYB* and *bHLH* regulatory genes in seedlings (e.g., *TT8*, *PAP1*, and *PAP2*; Dubos et al., 2008; Matsui et al., 2008) and in turn is activated by TT8, thus reducing the positive feedback loop of TT8 (Matsui et al., 2008).

MYBL2 also appears to mediate the response to environmental stresses and hormones. It is down-regulated by high light and up-regulated by low light, resulting in increased or reduced anthocyanin synthesis, respectively, through its regulation of *TT8*, *PAP1/PAP2* (Dubos et al., 2008; Rowan et al., 2009). *MYBL2* response to light depends on HY5 and occurs *via* three different mechanisms (Figure 3E). First, MYB-like Domain transcription factor (MYBD), which is induced in response to light and is a target gene of HY5, is a direct transcriptional repressor of MYBL2, able to reduce the acetylation of lysine 9 of histone 3 (H3K9) in MYBL2 promoter (Nguyen et al., 2015). Second, HY5 is also able to directly bind the *MYBL2* promoter and to repress its expression through an epigenetic mechanism, consisting of increased levels of H3K9 acetylation and H3K4 trimethylation at HY5 binding sites (Wang et al., 2016). Third, HY5 directly activates *miR858a*, which then inhibits MYBL2 by translational repression (Wang et al., 2016). Overall, *MYBL2* is negatively regulated by transcriptional repression by HY5 as well as by *miR858a via* translational repression (see also Section “Post-transcriptional Regulation of Anthocyanin Biosynthesis in Arabidopsis and Other Dicots”) and affects the expression of biosynthetic genes and anthocyanin accumulation through the interaction with MBW complexes. An additional level of epigenetic regulation involves the enrichment of the histone 2 variant H2A.Z in the promoter of anthocyanin biosynthetic genes in normal conditions as well as in high light and drought, in order to reduce an excessive anthocyanin accumulation. H2A.Z inhibits the expression of biosynthetic genes by reducing the accessibility and the H3K4 trimethylation around the transcription start site of genes (Cai et al., 2019).

Besides MYBL2, other transcription factors acting as repressors of anthocyanin biosynthesis in response to hormonal stimuli have been identified, including the JASMONATE ZIM-DOMAIN (JAZ) proteins (Qi et al., 2011) and the SQUAMOSA PROMOTER BINDING PROTEIN-LIKE 9 (SPL9; Gou et al., 2011). Despite their interactions with different proteins of the MBW complex, they share similar repressor mechanisms through ubiquitination and degradation by the 26S proteasome. JAZ proteins, which are the master regulators of jasmonic acid (JA) signaling, negatively regulate anthocyanin accumulation by interacting with bHLHs and R2R3-MYBs and disrupting the MBW complex (Figure 3C). Upon JA signaling, JAZ proteins are degraded through ubiquitination and degradation by the 26S proteasome and the reconstituted MBW complex activate anthocyanin biosynthesis (Qi et al., 2011). MYBL2 and JAZ proteins were also found to mediate gibberellic acid (GA)-inhibited anthocyanin biosynthesis in Arabidopsis (Xie et al., 2012, 2016). In the absence of GA, DELLA proteins, which are known repressors of GA signaling, are degraded (Jiang et al., 2007), but in the presence of GA they directly sequester MYBL2 and JAZ repressors, leading to the release of bHLH/MYB subunits and subsequently to the formation of an active MBW complex, which then activates the anthocyanin pathway. Possibly, a similar mechanism involves DELLA, MYBL2, and JAZs in response to abiotic stress-induced anthocyanin biosynthesis (Xie et al., 2012). Phosphate starvation and sucrose were also shown to reduce the concentration of GA or to specifically inhibit the GA-dependent degradation of DELLA proteins respectively, thus inducing anthocyanin accumulation in Arabidopsis (Hsieh et al., 2009). Similar to JAZ proteins, SPL9 acts as negative regulator of anthocyanin biosynthesis by interacting with R2R3-MYBs (PAP1 and MYB113) and disrupting the formation of the MBW complex with TT8 (Figure 3D; Gou et al., 2011; Cui et al., 2014).

Curiously, brassinosteroids (BR) negatively regulate the JA-induced anthocyanin accumulation by reducing the expression of *PAP1*, *PAP2*, and *GL3* (Peng et al., 2011). Whether this suppression is mediated by JAZ proteins or by MYBL2, which has been found to participate in the down-regulation of BR-repressed genes, is presently unknown (Ye et al., 2012). Furthermore, ethylene inhibits anthocyanin accumulation induced by sucrose and light by suppressing the expression of transcription factors that positively regulate anthocyanin biosynthesis, including *GL3*, *TT8*, and *PAP1*, while activating the negative regulator *MYBL2* (Jeong et al., 2010).

A NAC-type transcription factor ANAC032 has been proposed as an indirect negative regulator of the expression of LBGs (i.e., *DFR*, *ANS/LDOX*) and *TT8* regulatory gene under stress conditions (i.e., sucrose treatment, high light, and oxidative stress) through activation of the negative regulators of anthocyanin biosynthesis, MYBL2 and SPL9 (Mahmood et al., 2016). Furthermore, three members of the LATERAL ORGAN BOUNDARY DOMAIN (LBD) protein family (LBD37, 38, and 39) are strongly induced by nitrogen and function as transcriptional repressor of *PAP1* and *PAP2*, thereby suppressing anthocyanin biosynthesis in response to nitrogen (Zhou et al., 2012). Very recently, it has been highlighted the signaling pathway of strigolactones, which are carotenoid-derived hormones known to increase anthocyanin biosynthesis (Wang et al., 2020). More specifically, strigolactones trigger ubiquitination and degradation of SMAX-like 6 from Arabidopsis (AtSMXL6), a repressor interacting with PAP1, which is then released and allowed to activate anthocyanin biosynthesis (Wang et al., 2020).

Overall, the negative regulators mediating hormonal and environmental response (i.e., MYBL2, JAZ, and SPL9) act through a passive repression which disrupts the MBW complex by interacting with bHLHs (i.e., MYBL2, Figure 3B), with MYBs (i.e., SPL9, Figure 3D) or both (i.e., JAZ, Figure 3C). MYBL2 represents an exception, since it also actively represses transcription as part of the MBW complex (Figure 3B). Anthocyanin activation in response to hormones and abiotic stresses then occurs through a common mechanism, which involves the release of one or more of these negative regulators from the interaction with MYBs and/or bHLHs (Figures 3B–D), thus restoring a functional MBW complex that activates LBGs (Figure 3E).

Finally, some post-translational modifications of MBW proteins have been identified that negatively modulate the activity of MBW complexes. PAP1/PAP2, TT8/GL3/EGL3, and TTG1 are all short-lived and degraded by ubiquitin/26S proteasome (UPS)-dependent proteolysis (Maier et al., 2013; Patra et al., 2013a,b). For GL3/EGL3, the UPS targeting for degradation is part of a negative regulatory feedback loop likely functioning to reduce their hyperactivation (Patra et al., 2013b). For PAP1/PAP2, such degradation occurs in the dark and is mediated by the CONSTITUTIVE PHOTOMORPHOGENIC1 (COP1)/SPA E3 ligase, indicating that the light-regulated activation of PAP1/PAP2 is in part due to protein stabilization (Maier et al., 2013). A recent study has revealed that MAP-Kinase 4 (MAPK4) phosphorylation of PAP1 also increases its stability and is essential for light-induced anthocyanin accumulation (Li et al., 2016).

## Activators and Repressors of Anthocyanin Biosynthesis in Fruits, Vegetables, and Other Cereals

Transcription factors orthologous to the MBW complex of maize and Arabidopsis have been isolated in cereals and many fruits and vegetables, including grape, apple, kiwi, pear, peach and strawberry, with grape, apple, and tomato being the best studied in terms of mechanism of regulation of anthocyanin synthesis (Petroni and Tonelli, 2011; Jaakola, 2013; Chaves-Silva et al., 2018; Liu et al., 2018; Chen et al., 2019). Despite the fact that the MBW is highly conserved in fruits (Figure 2D), some *R2R3-MYBs*, such as *VvMYBF1* in grapevine and *SlMYB12* in tomato, are known that control the flavonol synthesis without a bHLH partner and are activators of EBGs (Czemmel et al., 2009; Ballester et al., 2010).

In rice, the *Purple leaf* (*Pl*) complex locus, including the *bHLH OsB1* and *OsB2* genes, was proposed to control the anthocyanin biosynthesis in rice leaves (Sakamoto et al., 2001). However, recent studies determined that the predominant MBW complex activating anthocyanin biosynthesis in purple-leaf rice includes the R2R3-MYB OsC1, the bHLH OsRb, and the WD40 OsPAC1, which contributes to the full activation of anthocyanin biosynthesis, similar to the maize *PAC1* gene (Figure 2D). Consistent with this, *OsRb* was found to be much higher expressed in leaves compared to *OsB1* and *OsB2*, whose expression level was extremely low (Zheng et al., 2019). Instead, *OsB2* and *OsC1* (also named *Kala4* and *Kala3*, respectively) have a predominant role in anthocyanin pigmentation of rice pericarp in black-grained rice (Maeda et al., 2014; Oikawa et al., 2015; Sun et al., 2018; Zheng et al., 2019) and are found to be negatively regulated by ethylene in the absence of light (Kumar et al., 2019), whereas the *bHLH* gene, *OsRc*, controls proanthocyanidin synthesis in pericarp of red rice varieties (Sweeney et al., 2006). Another *R2R3-MYB* gene, *OsP1*, closely related to the maize *P1* and the Arabidopsis *MYB11*, *MYB12*, and *MYB112*, was recently identified in rice. Similar to maize, OsP1 specifically activates a subset of anthocyanin biosynthetic genes (i.e., *CHS*, *CHI*, and *F3H*), with partial functional overlap with the rice MBW (Zheng et al., 2019). In other cereals, such as wheat, only the *Pp* (*Purple pericarp*) gene controlling anthocyanin pigmentation in seeds has been identified so far (McIntosh et al., 2003).

In grapevine, a complex locus with two *MYB* genes (*VvMYBA1* and *VvMYBA2*), homologs of Arabidopsis *PAP1*, *PAP2*, *MYB113*, and *MYB114* is specifically expressed in ripening berries, where it promotes the synthesis of tri-hydroxylated anthocyanins (i.e., delphinidin, malvidin, and petunidin), whereas the closely related *VvMYBA5*, *VvMYBA6*, and *VvMYBA7* are predominantly expressed in vegetative tissues and almost exclusively promote the synthesis of di-hydroxylated anthocyanins (i.e., cyanidin and peonidin; Azuma et al., 2008; Matus et al., 2017). Consistently, these regulators share the activation of late biosynthetic and modification/transport-related genes, but only VvMYBA1 and VvMYBA2 activate *F3′5′H* leading to tri-hydroxylated anthocyanins. In addition, *VvMYBA1* in fruits and *VvMYBA6-A7* in both plantlets and fruits were found to be responsive to UV-B in an HY5-dependent manner, similar to *PAP1* in Arabidopsis (Matus et al., 2017). The existence of an MBW complex is suggested by the identification of VvWDR1 as an activator of anthocyanin synthesis when overexpressed in Arabidopsis (Matus et al., 2010) and by the interaction of VvMYC1 with all grape MYBs inducing anthocyanins (i.e., VvMYBA1/A2/A6/A7), proanthocyanidins (i.e., VvMYBPA1/PA2; Matus et al., 2017), or both (i.e., VvMYB5a/5b; Figure 2D; Hichri et al., 2010). Similar to *TT8* in Arabidopsis, *VvMYC1* is involved in a positive feedback regulation of its own expression (Hichri et al., 2010). A retrotransposon insertion in the promoter of *VvMYBA1* (Kobayashi et al., 2004) and point mutations in *VvMYBA2* coding regions (Walker et al., 2007) are present in some white cultivars.

In apple, allelic homologs of *R2R3-MYBs* are responsible for the light-dependent anthocyanin pigmentation of red-skin apple cultivars (i.e., *MdMYB1* and *MdMYBA*; Takos et al., 2006; Ban et al., 2007) or determine red pigmentation in apple fruit and leaves (i.e., *MdMYB10*; Espley et al., 2007). MdMYB1 protein is degraded in the dark by the UPS, suggesting that the light-mediated anthocyanin accumulation in apple skin is due, at least in part, to the stabilization of these factors in light, similar to the Arabidopsis PAP1/PAP2 (Li et al., 2012c). On the other hand, the *MdMYB10* expression results from an autoregulatory loop mediated by five tandem repeats of an *MdMYB10* binding motif in its promoter (Espley et al., 2009). Other *R2R3-MYBs* activating anthocyanins are *MdMYB3* (Vimolmangkang et al., 2013), *MdMYB9*, and *MdMYB11*, also controlling proanthocyanidins (An et al., 2015), and *MdMYB110a*, a paralog of *MdMYB10* resulting from a whole-genome duplication event (Chagné et al., 2013). In apple, the MBW complex includes two bHLHs, MdbHLH3 and MdbHLH33, interacting with MdMYB1, MdMYB9, MdMYB10, and MdMYB11 (Espley et al., 2007; Xie et al., 2012; An et al., 2015) and an apple WD40 protein, MdTTG1, able to interact with bHLH proteins, but not MYBs (Brueggemann et al., 2010; An et al., 2012).

Interestingly, the apple *R2R3-MYBs* appear to regulate anthocyanin biosynthesis in response to low temperature and plant hormones through transcriptional and post-translational mechanisms similar to those highlighted in Arabidopsis. Upon exposure to low temperature, MdbHLH3 is transcriptionally activated and, once translated, the MdbHLH3 protein is phosphorylated to further enhance its transcription activation of MdMYB1, and form with it the MBW complex able to activate the genes of the anthocyanin biosynthetic pathway (Xie et al., 2012). On the other hand, MdbHLH3 protein was found to be sequestered by JAZ proteins, which are then degraded following JA exposure, allowing MdbHLH3 to act as direct activator of *MdMYB9* and *MdMYB11* and to form with them an MBW complex for activation of anthocyanin biosynthesis (An et al., 2015). Similarly, MdMYB10 is sequestered by the auxin-responsive factor MdARF13, which destabilizes the MBW complex and indirectly inhibits the anthocyanin biosynthesis, but it is also able to directly repress anthocyanin biosynthetic genes. Specifically, under low auxin concentration, the MdIAA121 repressor interacts with MdARF13 and prevents its binding to promoters of anthocyanin biosynthetic genes or to MdMYB10, whereas under high auxin concentration MdIAA121 is degraded by the 26S proteasome, thus allowing MdARF13 to repress anthocyanin biosynthesis (Wang et al., 2018). Finally, MdMYB1 was found to interact with the promoter of *ETHYLENE RESPONSE FACTOR3* (*ERF3*), a key regulator of ethylene biosynthesis, thus providing a positive feedback on ethylene biosynthesis and as a consequence an increase in anthocyanin accumulation (An et al., 2018).

Homologs of apple *MdMYB10* have been isolated in many other *Rosaceae*, including *PpMYB10*, *FaMYB10*, *PyMYB10*, and *PavMYB10.1* from peach, strawberry, pear, and cherry, respectively (Feng et al., 2010; Lin-Wang et al., 2010; Jin et al., 2016). In most of these fruits, an MBW complex has been identified. In peach (*Prunus persica*), PpMYB10, PpbHLH3, and PpWD40 are involved in the regulation of anthocyanin biosynthesis by forming a MBW complex (Figure 2D; Liu et al., 2015). In red-fleshed peaches, *PpMYB10* is activated by the NAC transcription factors BLOOD (BL) and PpNAC1, which are under control of the PpSPL1 repressor (Zhou et al., 2015). In addition, low temperature-dependent DNA demethylation has been recently observed in the promoter of anthocyanin biosynthetic genes, except for *PpGST* (Zhu et al., 2020). Instead, the expression and activity of PpGST, directly activated by PpMYB10 and PpbHLH3, were found to be a key step for anthocyanin regulation in a red-fleshed peach cultivar, resulting in a reduction of both anthocyanin accumulation and expression of biosynthetic and regulatory genes upon *PpGST* silencing (Zhao et al., 2020). In strawberry, FaMYB9/FaMYB11, FabHLH3, and FaTTG1 from the octoploid *Fragaria ananassa* form an MBW complex specifically activating proanthocyanidins (Schaart et al., 2013), whereas in a transient assay FvMYB10 and FvbHLH33 from the diploid *Fragaria vesca* were found to activate the anthocyanin-specific *DFR* and *UFGT* promoters (Lin-Wang et al., 2014). Recently, a RAV (related to ABI3/viviparous 1) transcription factor, FaRAV1, has been demonstrated to stimulate anthocyanin accumulation both by up-regulating *FaMYB10* and by directly binding to promoters of anthocyanin biosynthetic genes (Zhang et al., 2020a). In pear, an MBW complex has not been identified, but PyMYB114 and PybHLH3 were found to interact with PyERF3 in red pear fruit. In addition, PyMYB10 and PyMYB114 showed an additive effect in activating anthocyanins when co-expressed in tobacco and strawberry (Yao et al., 2017).

In kiwifruit, *AcMYB10* and *AcMYB110* from *Actinidia chinensis* activate the *CHS*, *DFR*, *flavonoid-3-galattosyltransferase* (*F3GT*) and *LDOX* genes (Li et al., 2017a; Herath et al., 2020), but the regulation of the branch points *F3′H* and *F3′5′H*, which determine the production of cyanidin- or delphinidin-based anthocyanins, respectively, has been attributed to *MYBC1* and *WRKY44*, which are also activators of the biosynthetic genes leading to proanthocyanidins (i.e., *FLS*, *LAR*, and *ANR*; Peng et al., 2020). In the proposed model, the MBW complex including MYB110 activates *CHS*, *DFR*, and *F3GT*, but when MYBC1 interacts with bHLH, WD40, and WRKY44, an MBWW complex (Figure 2D) activates the *F3′H* and *F3′5′H* genes, allowing both the anthocyanin and proanthocyanidin synthesis (Peng et al., 2020). Recently, *AcMYB123* and *AcbHLH42* have been also identified as activators of *ANS*, *F3GT* and anthocyanin biosynthesis in the inner pericarp of *A. chinensis* (Wang et al., 2019).

In tomato, two tightly linked *R2R3-MYB* genes, *SlANT1* and *SlAN2*, were considered as the main positive regulators controlling anthocyanin levels in the skin of fruits (Mes et al., 2008; Sapir et al., 2008). Both activated anthocyanin biosynthesis when overexpressed in tomato fruits, leaves, and flowers (Mathews et al., 2003; Schreiber et al., 2012; Meng et al., 2015), but only *SlAN2* was shown to act as a positive regulator of anthocyanin synthesis in vegetative tissues under high light or low temperature conditions through the control of the expression of the *bHLHs SlAN1* and *SlJAF13* (Kiferle et al., 2015). Expression of the *WDR* gene *SlAN11* seems to be constitutive and does not require *SlAN2*, whereas the expression of *SlANT1* is not detectable in wild-type tomato (Kiferle et al., 2015). This is consistent with studies in other species showing that MYB proteins regulate the transcription of their bHLH partners and subsequently form with the bHLH protein and the ubiquitously expressed WDR one an MBW complex that activates the anthocyanin genes. Recent studies have highlighted that the dominant *ANTHOCYANIN FRUIT (Aft)* gene, which was introgressed in cultivated tomatoes from the wild species *Solanum chilense*, co-segregates not only with *SlAN2* and *SlANT1* (Mes et al., 2008; Sapir et al., 2008), but also with two other *R2R3-MYB* genes, named *SlAN2like* and *SlANT1like* (Sun et al., 2019; Colanero et al., 2020; Yan et al., 2020). These studies proved that (i) only *SlAN2like* is necessary for anthocyanin synthesis (Sun et al., 2019); (ii) in cultivated wild-type tomatoes splicing mutations in *SlAN2like* lead to aberrant mRNAs producing shorter proteins lacking the most R2R3-MYB domain and the C-terminal domain, which are unable to interact with bHLH proteins in the MBW complex (Colanero et al., 2020), and (iii) the recently characterized CPC-like R3-MYB repressor ATROVIOLACEA (ATV) competes with SlAN2like in binding the bHLH SlAN1, thus contributing to a negative feedback loop that fine-tunes excessive anthocyanin synthesis (Colanero et al., 2018; Sun et al., 2019).

In other *Solanum* species, such as eggplant, pepper, and potato, no differences were observed in the expression level of *WD40* genes (i.e., *SmWD40*, *CaTTG1*, and *StAN11*, respectively) in pigmented compared to non-pigmented tissues, whereas the expression of *MYB* and *bHLH* genes was correlated to anthocyanin accumulation (Stommel and Dumm, 2015; Liu et al., 2016; Tang et al., 2020). Despite different *R2R3-MYB* putative activators have been reported in eggplant (*Solanum melongena*), only SmMYB1 and SmMYB75 have been demonstrated to activate anthocyanin biosynthesis in the fruit and to interact with SmTT8 in activating *SmCHS* (Zhang et al., 2016; Shi et al., 2021). In potato (*Solanum tuberosum*), the R2R3-MYB transcription factor StAN1 and the bHLHs StbHLH1 or StJAF13 are necessary for anthocyanin biosynthesis in tuber skin and flesh (Payyavula et al., 2013; Liu et al., 2016). In addition to StAN1, StMYBA1 and StMYB113 were also found to activate anthocyanin biosynthesis in tobacco by transient assays (Liu et al., 2016). Very recently, a genome-wide association study has identified two closely related *R2R3-MYB* genes, *StMYB88* and *StMYB89*, representing potential regulators of anthocyanin biosynthesis in tuber flesh (Li et al., 2021b). In pepper, the upregulation of *CaMYBA* and *CabHLH* has been correlated with the expression of anthocyanin biosynthetic genes in anthocyanin-pigmented fruits (Ohno et al., 2020). Recently, the *R2R3-MYB CaANT1*, *CaANT2*, and *CaAN1* genes have been proposed to interact with CaTTG1 in an MMBW complex able to regulate the expression of LBGs in purple pepper (Tang et al., 2020).

In carrot (*Daucus carota*), the R2R3-MYB transcription factor DcMYB7 has been demonstrated the main positive regulator of anthocyanin biosynthesis in purple roots by activating *DcbHLH3* and the biosynthetic genes (Iorizzo et al., 2019; Xu et al., 2019). Despite the overexpression of *DcMYB6* resulted in anthocyanin accumulation in Arabidopsis (Xu et al., 2017a), its overexpression in carrot did not determine anthocyanin accumulation in roots and high transcript levels were found in both pigmented and non-pigmented roots (Xu et al., 2019). Recently, DcMYB113 was found to activate *DcbHLH3* and anthocyanin biosynthetic genes, including those responsible for anthocyanin glycosylation and acylation (Xu et al., 2020). Other *MYB*, *bHLH*, and *WD40* genes have been associated with anthocyanin accumulation in carrot roots, but their functional validation is presently lacking (Kodama et al., 2018).

Similar to Arabidopsis, many subgroup 4 R2R3-MYB repressors have been identified in fruit species (Chen et al., 2019; LaFountain and Yuan, 2021). Based on phylogenetic analyses, these R2R3-MYBs are divided into AtMYB4-like repressors, which control the phenylpropanoid metabolism by directly inhibiting biosynthetic genes, and FaMYB1-like repressors, which regulate anthocyanin biosynthesis by disrupting the MBW complex (Chen et al., 2019; LaFountain and Yuan, 2021). FaMYB1 from strawberry is the first identified R2R3-MYB containing an EAR motif in the C-terminal domain in fruit species. Since then, other FaMYB1-like proteins acting upon MBW complexes have been identified. The *FaMYB1* gene is expressed at high levels only at ripe fruit stages, consistent with its role of anthocyanin repressor in the latter stages of strawberry fruit maturation (Aharoni et al., 2001; Lin-Wang et al., 2010). PhMYB27, and PhMYBx from petunia were found to act upon MBW complexes to repress anthocyanin synthesis (Koes et al., 2005; Albert et al., 2014), and similar mechanisms have been shown for FaMYB1, PpMYB18, SmMYB86, VvMYBC2, and VvMYB114 (Paolocci et al., 2011; Cavallini et al., 2015; Tirumalai et al., 2019; Zhou et al., 2019; Li et al., 2021a). A newly identified anthocyanin R2R3-MYB repressor, StMYB44, containing an EAR-repression domain, represses the anthocyanin biosynthesis in tuber flesh in response to high temperature without interacting with a bHLH factor. In fact, StMYB44 down-regulates the expression of *StAN1*, *StbHLH1*, and anthocyanin biosynthetic genes, thus resulting in a redirection of metabolic flux into chlorogenic acid or lignin biosynthesis (Liu et al., 2019). Interestingly, the *R2R3-MYB27* repressor from kiwifruit is transcriptionally inhibited by high sugar concentrations (i.e., trehalose 6-phosphate), whereas it is consistently up-regulated upon carbon starvation, and has been proposed to mediate the reduced anthocyanin accumulation in red-fleshed fruits in response to sugar depletion (Nardozza et al., 2020).

A VvMYB4-like transcription factor from grape, highly expressed in the skin of berries, resulted in down-regulation of LBGs when overexpressed in tobacco flowers (Pérez-Díaz et al., 2016). As an exception, the AtMYB4-like MdMYB16 acts as homodimer to directly inhibit the anthocyanin biosynthetic genes *via* its EAR repressor domain (Xu et al., 2017b), but may also be able to weaken the interaction of MdMYB10-MdbHLH3/MdbHLH33 complex acting on anthocyanin genes (Lin-Wang et al., 2010).

## Post-Transcriptional Regulation of Anthocyanin Biosynthesis in Arabidopsis and Other Dicots

In recent years, several studies have highlighted the important role of miRNAs involved in fine-tuning flavonoid biosynthesis in Arabidopsis. Based on initial studies and recent validations, *MYB11*, *MYB12*, and *MYB111* have been confirmed as targeted by *miR858* in Arabidopsis (Figure 3E), thereby regulating flavonol production (Sharma et al., 2016; Liu et al., 2021). On the other hand, the expression of *TCP3*, and therefore its activity as anthocyanin enhancer through the interaction with the MBW complex and the MYBL2 repressor (Figure 3A), is feedback inhibited by *miR319* (Nag et al., 2009).

In Arabidopsis, SPL9, which negatively regulates anthocyanin biosynthesis through interfering with the formation of the MBW complex, is also under the negative control of *miR156* and determines the acropetal accumulation of anthocyanins in Arabidopsis stem. Increased *miR156* activity at the junction between rosette and stem promotes high levels of anthocyanins, whereas reduced *miR156* activity in the upper part of stem results in SPL9 repression of anthocyanin synthesis and redirection of metabolic flux to high levels of flavonols (Gou et al., 2011). In addition, when *miR156* is induced by salt and drought stress, *SPL9* is repressed, resulting in the activation of the anthocyanin pathway through PAP1 activity (Figure 3E), whereas in non-stressed conditions, *miR156* is suppressed and SPL9 repression of anthocyanins is restored (Cui et al., 2014). In grape, *miR156* expression was found to be modulated by multiple hormonal signals (i.e., induced by JA and abscisic acid, ABA; repressed by GA and auxin) and to modulate anthocyanin synthesis through SPL9 (Su et al., 2021).

Several studies showed that *miR828* is also involved in the negative regulation of anthocyanin biosynthesis in response to different signals (Hsieh et al., 2009; Luo et al., 2012; Yang et al., 2013). Sucrose, phosphate starvation, and ABA have been shown to directly induce *Trans-acting siRNA gene 4 (TAS4)* and *miR828* and indirectly through activation of *PAP1*, *PAP2*, and *MYB113* expression. *TAS4* cleavage is then triggered by *miR828* into *TAS4-siR81(−)*, which in turn down-regulates *PAP1*, *PAP2*, *MYB113*, and *MYB82* in a regulatory feedback loop that fine-tunes anthocyanin biosynthesis (Figure 3E). Interestingly, *miR828* also directly targets *MYB113*, suggesting a close relationship between these MYBs, *miR828*, and *TAS4* (Hsieh et al., 2009; Luo et al., 2012; Yang et al., 2013). Similarly, *miR828* negatively controls anthocyanin biosynthesis by repressing the expression of *BrPAP1*, *BrPAP2*, and *BrMYB82* through *BrTAS4* in *Brassica rapa* (Zhou et al., 2020). In apple, *Md-TAS4-siR81(−)* is activated by MdMYB1 during the late fruit maturation stage and in response to high temperature and reduces anthocyanin biosynthesis by targeting *MdbHLH3* (Zhang et al., 2020b).

In contrast, in Arabidopsis *miR408* and *miR858a* positively regulate anthocyanin biosynthesis in seedlings. HY5 and SPL7 have been found to co-regulate several genes in response to light and copper, respectively, including anthocyanin biosynthetic genes through coordinate activation of *miR408*, which promotes anthocyanin accumulation through an undefined mechanism (Zhang et al., 2014b). As previously reported, *miR858a* has also been demonstrated to enhance anthocyanin biosynthesis in seedlings by inhibiting the expression of MYBL2 through translational repression (Wang et al., 2016). *miR858* together with *miR828* inhibits the anthocyanin repressor *VvMYB114* in grape resulting in the activation of anthocyanin biosynthesis (Tirumalai et al., 2019). However, in kiwifruit and tomato, *miR858* negatively regulates the anthocyanin pathway by repressing *AaMYBC1* (Li et al., 2019) or by modulating *SlMYB48-like* and *SlMYB7-like* putative activators, respectively (Jia et al., 2015).

Although the mature sequence of *miR858* is very similar, if not identical, in different species, these findings indicate that *miR858* is associated with negative regulation in tomato and kiwifruit and positive regulation in Arabidopsis. Similarly, in grape miR828 and TAS4-siR81(−) activate anthocyanin biosynthesis by inhibiting a MYB repressor, whereas in Arabidopsis, *B. rapa* and apple they down-regulate anthocyanin accumulation by inhibiting MYB repressors or bHLH proteins. Overall, this suggests that the miR858/miR828-mediated mechanisms underlying anthocyanin biosynthesis may be distinctive in different species and depend on the downstream targets affected.

Interestingly, two long noncoding natural antisense RNAs (lncNATs), named *asDcMYB6* and *asDcMYB7*, have been shown to be transcribed in opposite direction to *DcMYB6* and *DcMYB7*, respectively, and to be highly expressed in purple carrots compared to orange ones, like their corresponding genes. These lncNATs may represent a novel player in post-transcriptional regulation of anthocyanin biosynthesis in carrot (Chialva et al., 2021).

## Enhancing Anthocyanin Content in Our Plant Food

The health benefits of anthocyanins make them important targets for improving existing commercial varieties for anthocyanin-rich functional foods, which help consumers to achieve a greater content of anthocyanins in their diets. Enrichment of bioactives could be particularly useful to assess the nutritional properties of different bioactives by comparing near-isogenic plant-based foods that vary only in the type and quantity of the bioactives under analysis (Martin et al., 2011).

Most efforts have been focused on increasing anthocyanins by introducing or inducing the expression of MBW transcription factors to activate the endogenous anthocyanin biosynthetic genes either by conventional breeding or by metabolic engineering.

The introgression of dominant mutations (i.e., *Aft* and *Aubergine*, *Abg*) through interspecific crosses with wild species transferred the ability to produce anthocyanins in the peel of cultivated tomatoes (Jones et al., 2003; Mes et al., 2008). Furthermore, the recessive gene *atroviolacea* (*atv*) and the constitutive photoresponsive *high-pigment-1* (*hp-1*) allele in combination with *Aft* or *Abg* have been shown to stimulate a higher production of anthocyanins in the peel (Povero et al., 2011; Catola et al., 2017). Similarly, taking advantage of geographic accessions of the *MYB* and *bHLH* regulatory gene families (*C1/Pl1* and *B1/R1*, respectively) from Andean corn cultivars, maize hybrids carrying different levels of anthocyanins in kernels have been obtained (Petroni et al., 2014).

In order to increase the anthocyanin content in tomato fruits, anthocyanin regulatory genes from different plant species have been expressed in tomato most successfully when the *bHLH Delila* and *MYB Rosea1* genes from snapdragon were overexpressed under the control of a tomato fruit-specific promoter, to give purple tomato fruits containing large amounts of anthocyanins (i.e., delphinidin) and flavonols (i.e., myricetin) both in the peel and flesh (Butelli et al., 2008). High expression of *AtMYB12*, controlling flavonol synthesis in Arabidopsis, together with *Delila* and *Rosea1* further enhanced anthocyanin production in tomato by activating pathways of primary metabolism (glycolysis, the TCA cycle, the oxidative pentose phosphate pathway and the shikimate pathway) toward the production of substrates, ATP and reducing power for the phenylpropanoid pathway (Zhang et al., 2015). A similar strategy was used to obtain tomato fruits with high levels of resveratrol and genistein, reaching levels comparable to those present in soy-based products, like tofu (Zhang et al., 2015). Based on a similar concept, arogenate dehydratases (*ADT*) genes, controlling the level of phenylalanine, have been proposed as new targets for metabolic engineering to modulate anthocyanin content in plants (Chen et al., 2016). In general, metabolic engineering that combines multi-level transcriptional regulation and pathway rerouting offers an excellent strategy for biofortification of foods, for the production of plant-derived phytochemicals and ingredients, and for establishing materials for comparative nutrition studies. Such comparisons should lead to much clearer understanding of the health benefits of foods rich in specific polyphenolic phytonutrients in the diet, and shed light on their mechanisms of action (Zhang et al., 2015; Fu et al., 2018).

Novel regulatory targets for enhancing anthocyanin biosynthesis in plant food could include the COP1-mediated degradation of MYB transcription factors involved in anthocyanin production, such as MdMYB1 in apple (Li et al., 2012c), which could provide a very efficient and specific approach to increasing anthocyanin levels while maintaining otherwise normal light signaling. Alternatively, the control of expression of MYB transcription factors by small RNAs, like *miR828*/*TAS4* and *miR858* or other negative regulators, such PpSPL1 in peach (Zhou et al., 2015) and R2R3-MYB repressors found to reduce anthocyanin biosynthesis in fruits and vegetables, might be exploited to increase anthocyanin synthesis in our foods, perhaps using genome editing techniques. Beyond this, an understanding of the mechanisms that determine anthocyanin stability, such as anthocyanin decoration (i.e., glycosylation and acylation) or pH in vacuoles, as well as the fading processes operational in some flowers, has been suggested as additional tools for both biotechnological approaches and marker-assisted breeding (Zhang et al., 2015; Passeri et al., 2016).

## Assessing Health Benefits of Anthocyanin-Rich Functional Foods

There are several reviews of epidemiological and preclinical intervention studies reporting the beneficial effects of anthocyanins on health (He and Giusti, 2010; Tsuda, 2012; Pojer et al., 2013; Wallace, 2013; Wallace and Giusti, 2015; Li et al., 2017b; Mattioli et al., 2020; Salehi et al., 2020; Kozłowska and Dzierżanowski, 2021). Despite this, in developed countries, there is an increasing trend in eating energy-dense foods, rich in sugar and saturated fatty acids. The consumption of fruits and vegetables has declined substantially over the past 30years, with a consequent increase in obesity, especially childhood obesity, defined as a body mass index (BMI) ≥30 (Martin et al., 2011). Increased consumption of anthocyanin-rich fruits and vegetables could have positive effects on health. This is even more important considering that purified anthocyanins consumed as dietary supplements do not have the same beneficial effects as anthocyanins in a natural food matrix (Prior et al., 2008; Titta et al., 2010; Martin et al., 2011). In this context, the development of near-isogenic genotypes of common foods, devoid or rich in anthocyanins is of importance for four reasons. First, the production of near-isogenic plant foods allows to reduce some of the complexity of food in the diet–health relationship and provide model foods that can be used for both animal feeding studies and human intervention trials for assessing the role of plant bioactives in the diet. Specifically, being near-isogenic, the anthocyanin-free genotypes represent a matched control for assessing the health-protective effects of anthocyanins, allowing the identification of their specific mechanisms of action compared to those of other polyphenols and phytonutrients present in the food matrix. Second, different anthocyanin-rich foods can be used to assess whether the consumption of comparable amounts of anthocyanins in different food matrices gives the same health benefits against different specific diseases. Third, anthocyanin-rich foods can be used in in animal models or directly in human intervention studies, if there are no safety concerns, to validate their health benefits. Fourth, once assessed, they can add back health-promoting anthocyanins to the diet. Anthocyanin-rich and anthocyanin-free comparator foods have been developed successfully either by conventional breeding (i.e., anthocyanin-rich corn and orange) or by metabolic engineering (i.e., anthocyanin-rich tomato and apple) and used to test the health benefits of anthocyanins in animal models and in some pilot intervention studies. Below we provide a summary of their beneficial effects (also reported in Table 1).

**Table 1:** Health effects of anthocyanin-containing foods against anthocyanin-free comparators.

Functional food	Form of administration	Target	Experimental model	Effects	References
Anthocyanin-rich maize	Blue and yellow corn in rodent diet	Heart	Wild-type rats	Reduced infarct size and improved antioxidant defenses	Toufektsian et al., 2008
	Blue and yellow corn in rodent diet	Heart	Wild-type rats	Increased plasma concentration of DHA and EPA	Toufektsian et al., 2011
	Purple and yellow corn in rodent diet	Heart	Wild-type mice	Protect against Doxorubicin adverse side-effects	Petroni et al., 2017
	Purple corn extract in rodent diet vs. not-supplemented diet	Obesity	Wild-type mice	Prevent body weight gain under HFD	Tsuda et al., 2003
	Purple corn extract in rodent diet vs. not-supplemented diet	Obesity	Wild-type mice	Attenuate HFD-induced inflammation with a long-lasting reprogramming of ATM toward an anti-inflammatory status	Tomay et al., 2019
	Purple and yellow corn extract in rodent diet	Brain	Wild-type rats	Reduced allodynia and neuroinflammation	Magni et al., 2018
	Purple and yellow corn in rodent diet	Muscular dystrophy	*Sgca* null mice	Delayed progression of muscular dystrophies reducing inflammation and oxidative stress	Saclier et al., 2020
	Purple and yellow corn in rodent diet	Liver	Wild-type mice	Regulated H3K4me3 affecting specific pathways	Persico et al., 2021
	Purple corn extract in drinking water vs. water	Diabetes	*db/db* mice	Delayed diabetes-associated renal fibrosis and mesangial inflammation	Li et al., 2012a,b
	Purple corn extract in drinking water vs. water	Diabetes	*db/db* mice	Reduced diabetes-associated glomerular monocyte activation, macrophages infiltration and angiogenesis	Kang et al., 2012, 2013
	Blue and yellow corn in rodent diet	Brain	Wild-type rats	Reduced mtDNA damage	Demeilliers et al., 2017
	Purple corn extract in rodent diet vs. not-supplemented diet	Cancer	Wild-type rats	Delayed mammary cancer	Fukamachi et al., 2008
	Purple corn extract in rodent diet vs. not-supplemented diet	Cancer	Wild-type rats	Delayed progression of prostate cancer	Long et al., 2013
Anthocyanin-rich orange	Moro and Navelina orange juice	Obesity	Wild-type mice	Prevent body weight gain under HFD	Titta et al., 2010
	Moro and Navelina orange juice	Obesity	Wild-type mice	Prevent body weight gain and liver steatosis under HFD	Salamone et al., 2012
Anthocyanin-rich tomato	Purple and red tomato powder in rodent diet	Cancer	*p53^−/−^* mice	Delayed cancer development and increased life span	Butelli et al., 2008
	Bronze and red tomato powder in rodent diet	Inflammation and microbiota	Winnie mice	Reduced inflammation markers and modulated gut microbiota	Liso et al., 2018; Scarano et al., 2018
	Bronze and red tomato powder in rodent diet	Inflammation and microbiota in mothers and puppies	Winnie mice	Reduced inflammation markers and modulated gut microbiota	De Santis et al., 2021
Anthocyanin-rich apple	Red and white *near-isogenic* apple in rodent diet	Inflammation and microbiota	Wild-type mice	Reduced inflammation markers and modulated gut microbiota	Espley et al., 2014
	Naturally bred red and yellow-fleshed apple in human diet	Inflammation and microbiota	Healthy humans	Promoted immune function	Barnett et al., 2021
	Biofortified red and white fleshed apple in rodent diet	Hypercholesterolemia	Wild-type rats	Protect against HFD-induced cardiovascular and metabolic alterations	Yuste et al., 2021
	Naturally bred red and white fleshed apple in rodent diet	Cancer	Wild-type rats	Delayed appearance of the precancerous markers	Bars-Cortina et al., 2020

*HFD, high-fat diet; mtDNA, mitochondrial DNA; DHA, docosahexaenoic acid; EPA, eicosapentaenoic acid; ATM, adipose tissue macrophages; H3K4me3, trimethylation of lysine 4 of the histone H3*.

### Anthocyanin-Rich Maize

Anthocyanin-rich maize (i.e., blue and purple corn) originates from South America, where it is largely used also as colorant for food and beverages. It mainly contains cyanidin 3-glucoside and, to a small extent, pelargonidin 3-glucoside and peonidin 3-glucoside (Pedreschi and Cisneros-Zevallos, 2007; Petroni et al., 2017). The beneficial effects of purple corn have been recognized for a very long time. Aztecs used to prepare a beverage rich in purple corn extract, called *Tlaolli*, used to treat a number of illnesses.

There are indications that a daily intake of anthocyanins in quantities comparable to those consumed in a Mediterranean diet is protective against cardiac injuries and pathologies. The cardioprotective effects of an anthocyanin-rich diet were tested using an *ex vivo* model of isolated perfused rat heart (Toufektsian et al., 2008). The infarct size in rats fed with anthocyanin-rich *R1 C1* blue corn diet for 8weeks was reduced compared with those of rats fed with the near-isogenic *r1 c1* yellow corn diet, meaning that anthocyanins can induce a state of myocardial resistance. Moreover, the anthocyanin-rich diet was able to increase the total and reduced glutathione in preischemic heart, suggesting that the protection against ischemia-reperfusion injury might be related, at least in part, to an improvement in endogenous antioxidant defenses. Most importantly, dietary anthocyanins from blue corn were shown to modulate the metabolism of (n-3) polyunsaturated fatty acids (PUFA) and increase plasma concentrations of eicosapentaenoic acid (EPA) and docosahexaenoic acid (DHA), two fatty acids known to have a very important role of protection against heart disease complications (Toufektsian et al., 2011). Hence, the comparison of these isogenic corn lines showed that anthocyanins might exert their beneficial effects in two ways: directly, e.g., increasing the endogenous antioxidant defenses, and/or indirectly, as signaling molecules modulating other metabolic pathways.

Dietary anthocyanins from purple corn have been shown to be protective against the cardiotoxic side effects of chemotherapeutic drugs, like Doxorubicin (DOXO). A recent study (Petroni et al., 2017) demonstrated that mice fed with a purple corn-rich diet (Red diet, RD) were more resistant to DOXO-induced cardiac alterations (i.e., disorganized myofibrils, mitochondrial fragmentation/degradation, and defects in sarcolemma junctions) than mice fed with an isogenic yellow corn diet (YD). Moreover, the mid-term DOXO-induced mortality was significantly attenuated in mice on the RD compared to mice fed with YD. In addition, a purple corn extract did not interfere with the chemotherapeutic activity of DOXO in tumor cell lines. The mechanisms by which purple corn protects against DOXO side effects are still under investigation.

Dietary anthocyanins from blue corn have also been shown to be protective from brain mitochondrial DNA (mtDNA) damage induced by ethanol. Oxidative stress due to ethanol metabolism is known to cause damage to mtDNA. Rats, divided into 4 experimental groups, were fed for 8weeks with anthocyanin-rich or anthocyanin-free diets while receiving 12% ethanol or water as beverages. Mice consuming the anthocyanin-free diet and ethanol showed increased reactive oxygen species (ROS) and mtDNA damage in their brains, whereas consumption of ethanol with the anthocyanin-rich diet did not show the accumulation of damaged mtDNA. Again, this may be due to the induction of antioxidant defense responses promoted by the blue corn diets (Demeilliers et al., 2017).

Other studies have demonstrated that anthocyanins from purple corn have preventive effects on the development of obesity and hyperglycemia induced by the consumption of a high-fat diet (HFD). When fed for 12weeks with a HFD, addition of purple corn extracts to the diet prevented weight gain and hypertrophy of adipocytes, which is an increase in cell size of adipocytes generally associated with increased cellular stress in the adipose tissue and with systemic diabetes (Tsuda et al., 2003). Moreover, in cell cultures of human adipocytes, cyanidin 3-glucoside (C3G) positively regulated obesity and type 2 diabetes markers increasing adiponectin and down-regulating PAI-1 (Plasminogen Activator Inhibitor-1) and IL-6 (Interleukin-6; Tsuda et al., 2006). Adiponectin is the most important adipocytokine, and its expression is inversely correlated to the amount of fat tissue in the body: In conditions of obesity and type 2 diabetes, it is downregulated, while it is up-regulated during starvation (Kadowaki et al., 2006; Lee and Shao, 2006). Elevated levels of PAI-1 and IL-6 are characteristics of obesity and type-2 diabetes (Jung and Choi, 2014). Another study on the adipose-tissue macrophages (ATM) confirmed the protective effect of purple corn against obesity-related inflammation. Macrophages of mice fed with HFD and purple corn extract (HFD+RED) for 12weeks showed an M2 anti-inflammatory phenotype associated with increasing production of anti-inflammatory markers and tissue repair (i.e., *Arginase I*, *ArgI*; *Found in inflammatory zone 1*, *Fizz1*; *Transforming growth factor β*, *TGFβ*), while mice receiving HFD and water had M1 macrophages that produce pro-inflammatory cytokines and encourage inflammation and tissue destruction (i.e., *IL-6*; *Interleukin-1β*, *IL-1β*; *Tumor necrosis factor α*, *TNF-α*; *Cyclooxygenase-2*, *COX-2*). Moreover, adipose tissue M2 macrophages obtained from HFD+RED mice and treated with lipopolysaccharide (LPS) *ex vivo*, maintained the anti-inflammatory phenotype, indicating a long-lasting effect of anthocyanins (Tomay et al., 2019).

The ability of anthocyanins to exert antioxidant and anti-inflammatory activity makes them eligible for the study of different pathologies characterized by both oxidative stress and inflammation. A study conducted on Sgca null dystrophic mice, with a severe degenerative myopathy similar to Duchenne, has demonstrated that anthocyanins from purple corn counteracted the progression of muscular dystrophy (early and late-stage) acting on both oxidative and inflammatory status without affecting regeneration. Purple diet, but not the near-isogenic yellow diet, ameliorated tissue morphology, fibrosis, and muscle performance, promoted a metabolic shift to an oxidative fiber metabolism, and increased the mitochondrial amount counteracting the progression of the disease. Finally, mice fed with purple corn diet presented less macrophage infiltration compared with the yellow diet counterpart (Saclier et al., 2020).

Anthocyanins from purple corn reduced the orofacial pain induced by the inflammation of the trigeminal nerve, by preventing the macrophage infiltration in the trigeminal ganglion and the activation of microglia (i.e., macrophages resident in the nervous central system) both *in vivo* and *in vitro*. In a rat model of trigeminal sensitization drinking near-isogenic yellow or purple corn extract, anthocyanins and acetyl salicylic acid (ASA) equally reduced allodynia and macrophage infiltration, but only purple corn extract inhibited microglial activation *in vivo* and reverted LPS-induced inflammation *in vitro* resulting in lower production of pro-inflammatory mediators (IL-6, TNF-α, IL-1β; Monocyte Chemoattractant Protein-1, MCP-1; inducible nitric oxide synthase, iNOS) and in an increase in the anti-inflammatory ones (Interleukin-10, IL-10; Interleukin 13, IL-13; Arg-1; Fizz1, YM-1; Magni et al., 2018).

The nutriepigenetic effect of anthocyanins was also recently investigated. Anthocyanins, as many other phytonutrients, can alter the phenotype through epigenetic modifications. One of these modification includes histone tail modifications that can alter structure of the chromatin and modify gene expression and function. Many histone modifications have been identified, and the most studied one is the trimethylation of lysine 4 of histone H3 (H3K4me3), which is associated with transcribed genes in mice and humans. Persico et al. (2021) analyzed the effect of five different diets (standard, caloric restriction, high fat, purple corn, and yellow corn) on H3K4me3 in mice liver, highlighting that anthocyanins from purple corn regulated H3K4me3 affecting different pathways like the integrin-like kinase signaling, involved in inflammation, and the metabolism of the pyruvate and amino acids (Persico et al., 2021).

Purple corn extract can also ameliorate diabetes-associated diseases, retarding diabetic nephropathy, ameliorating hyperglycemia, counteracting renal filtration dysfunctions in *db/db* mice and inhibiting high-glucose-induced fibrosis and inflammation *in vitro* (Li et al., 2012a,b). Moreover, purple corn extract reduced the clinical manifestations of diabetic nephropathy, since it lowered diabetes-associated glomerular mesangial expansion (i.e., the accumulation of extracellular matrix proteins in the mesangial interstitial space) and macrophage infiltration into diabetic glomeruli (Kang et al., 2012) and counteracted glomerular angiogenesis (Kang et al., 2013), thus representing a potential complementary therapy for diabetes-associated glomerulosclerosis, inflammation, and angiogenesis.

There is some evidence that purple corn has anticancer activity as well. Model rats treated with a heterocyclic amine carcinogen showed that purple corn extract had an anti-tumor activity exerted through the modulation of cell proliferation and apoptosis in the mammary neoplastic lesions, due to the reduction of RAS protein level, which is commonly higher in tumors since it promotes cell growth through the Phosphatidyl Inositol 3-Kinase/Akt (PI3K/Akt) and Extracellular signal-regulated kinase 1/2 (Erk1/2) pathways. The decrease in RAS and phosphorylated Akt correlated with the increase of the cleaved caspase 3 which induced apoptosis (Fukamachi et al., 2008). More recently, purple corn extract was reported to retard the progression of the tumor, reducing the percentage of adenocarcinoma in a dose-dependent manner in Transgenic Rats for Adenocarcinoma of Prostate (TRAP) model rats. The most effective anthocyanins appeared to be both cyanidin 3-glucoside and pelargonidin 3-glucoside (Long et al., 2013).

### Anthocyanin-Rich Orange

Tarocco, Moro, and Sanguinello are the three major blood varieties that differ from the other varieties of the sweet orange group (Valencia Late, Washington navel, and Navelina) for the presence of anthocyanins, mainly represented by C3G. Sweet orange is an interspecific hybrid that has no sexual segregation, so that all varieties of this group can be considered near-isogenic (Butelli et al., 2017). Tarocco is from Italy, and it is medium-sized seedless and very flavorful. It is also called “half-blood” because the flesh is less red-pigmented than the other two varieties. The Moro oranges produce the highest levels of anthocyanins, and they are called “deep blood orange” and they originate from Italy. Sanguinello comes from Spain, but is also cultivated in Sicily; it is called “full-blood” orange and has similar characteristics to Moro (Grosso et al., 2013). All three blood varieties of sweet orange have a common ancestor, since they all carry the same *Copia*-like retrotransposon in the subgroup 6 *R2R3-MYB Ruby* gene, responsible for the cold-dependent fruit-specific activation of anthocyanin biosynthesis, and arose through selection of bud mutations (Butelli et al., 2012). Moro and the common sweet orange Navelina contain comparable concentrations of vitamin C, flavanones, and hydroxycinnamic acids, thus allowing their use in comparative nutritional studies with the aim of testing their obesity-preventing activities (Titta et al., 2010).

Dietary supplementation of Moro juice significantly reduced body weight gain and fat accumulation in mice. Mice fed for 12weeks with a standard diet (SD, 3.3kcal/g, mainly composed of carbohydrates) together with Moro juice, gained less weight than mice drinking water or Navelina juice. Moro juice was also effective in almost abolishing weight gain induced by a HFD (5.24kcal/g, with 60% fat supplement), reducing the abdominal and inguinal fat mass by approximately 50%, while showing a marked reduction in adipocyte cell size and lipid accumulation. Mice fed a HFD with drinking water or Navelina juice showed no such reduction (Titta et al., 2010). In addition, glucose, fatty acid, and triglyceride blood levels were not altered in mice drinking Moro juice on a HFD. Analysis of the transcriptomes of adipocytes of the mice on the different diets revealed that the Moro juice can counteract the effects of the HFD on adipocytes by altering gene expression. Indeed, the gene expression profiles of mice on the HFD regimen drinking Moro juice, but not of those drinking Navelina juice, were similar to those of mice fed with the SD, preventing the change in expression of 21% of the up-regulated and the 55% of the down-regulated genes in response to the HFD (Titta et al., 2010). In agreement with this study, mice fed with HFD and Moro juice showed a reduction in body weight gain compared to mice in the same condition but drinking water. Moreover, Moro juice could counteract liver steatogenesis in HFD fed mice (Salamone et al., 2012).

Analyses performed using Moro anthocyanin-rich extracts confirmed the beneficial effects in reducing fat accumulation in mice, but the Moro extract was less effective than crude Moro juice. The administration of purified C3G did not show any effect on weight gain (Titta et al., 2010). This suggested that other components of the Moro juice, in addition to anthocyanins, may contribute to the anti-obesity effects. For these reasons functional foods are extremely important and the consumption of fresh fruit has to be preferred to fruit extracts or fruit-derived supplementations.

A study in healthy human volunteers consuming either blood orange juice or blond juice, showed that consumption of either type of orange juice decreased the pro-coagulant activity of whole blood, an indicator of cardiovascular risk. This suggested a role of antioxidants independent of the anthocyanin content of blood orange juice (Napoleone et al., 2013). Another study on healthy humans showed potential protective effects of both blood and blond orange juice on the low-grade pro-inflammatory status induced by the consumption of a standardized fatty meal (Cerletti et al., 2015). Consumption of a fatty meal can induce an acute inflammatory status, defined by an increase in platelet and leukocyte counts and in myeloperoxidase (MPO) degranulation of granulocytes. Granulocytes release MPO, a peroxidase enzyme, into the extracellular space in the inflammatory locus, increasing inflammation. Frequent fatty meal consumption may lead to chronic low-grade inflammation and to a series of events that may develop into atherothrombosis (Cerletti et al., 2016). Consumption of both blood and blond orange juice prevented neutrophil MPO degranulation, used as a marker of cell activation induced by the fatty meal, but did not modify other leukocyte cellular markers. High anthocyanin, blood orange juice was effective in reducing total cholesterol in plasma, unaccompanied by high-density lipoprotein (HDL) changes. Both juices reduced blood glucose levels (Cerletti et al., 2015). Other preclinical studies have confirmed that consumption of blood oranges can have anti-inflammatory effects and limit body weight gain, enhance insulin sensitivity and decrease serum triglycerides and total cholesterol in mice (Grosso et al., 2013).

### Purple Tomato

Tomato is among the most important vegetables consumed world-wide. It is rich in vitamins, flavonoids, and other health-promoting compounds, but usually it does not contain anthocyanins, except in some tomato wild species, such as *S. chilense* (Jones et al., 2003). The red color, in fact, is due to the presence of carotenoids, including lycopene and the orange-colored β-carotene. Since tomato is the second most consumed among vegetables in the human diet, the importance of this fruit as a vehicle for nutrients and bioactive compounds for improving human health, is clear. Biotechnological and conventional breeding approaches have been used to engineer anthocyanin production in tomatoes (Gonzali et al., 2009).

Tomatoes genetically engineered to produce high levels of delphinidin and petunidin were produced through the expression of *Delila* and *Rosea1* regulatory genes from *A. majus*, specifically in fruit (Butelli et al., 2008). To investigate whether the anthocyanin levels reached were enough to promote health, diets supplemented with 10% red or purple tomato powder were fed to cancer-prone *Trp53^−/−^* mice. Mice lacking *p53* are prone to develop soft tissue carcinoma at an early age. Mice fed with purple tomato powder supplemented diets showed a significant extension of life span compared to mice fed diets supplemented with red wild-type tomato powder or SD without supplementation. This demonstrated that dietary consumption of high levels of anthocyanins can extend the life span of *Trp53^−/−^* cancer-prone mice by as much as 30% (Butelli et al., 2008).

Recent studies indicate an association between purple tomato and a reduction in the severity of symptoms of inflammatory bowel disease (IBD), a chronic inflammation of the gut including Crohn’s disease and ulcerative colitis (Liso et al., 2018; Scarano et al., 2018; De Santis et al., 2021). Tomato lines with different combinations of polyphenols have been tested in a mouse model of IBD, demonstrating that tomato enriched in flavonols, anthocyanins, and stilbenoids (named Bronze) were able to reduce/delay the symptoms as well as the dysbiotic intestinal microbiota associated with dextran sodium sulfate (DSS)-induced colitis, and showed significantly diminished pro-inflammatory mediators IL-6 and TNF-α levels. Interestingly, the combination of different polyphenols was more effective than single flavonoid classes (Liso et al., 2018; Scarano et al., 2018), and determined a reduction of mother’s dysbiosis and prevented/reduced IBD development in puppies, when supplied during pregnancy and lactation (De Santis et al., 2021). Overall, these studies indicate that tomato extracts enriched in multiple classes of flavonoids, including anthocyanins, display not only a direct anti-inflammatory role, but also a change on the gut microbiota that prevents a chronic inflammation status of the gut.

### Anthocyanin-Rich Apple

Apple and apple-related products are some of the most important dietary sources of polyphenols. Moreover, recent discoveries suggest that apple consumption reduces the risk of a number of chronic diseases (Boyer and Liu, 2004; Hyun and Jang, 2016). Anthocyanins accumulate preferentially in the peel, where they are responsible for the color of apples. In addition, some consumers are used to peel apples and other fruits before eating them, thus limiting the consumption of anthocyanins from these dietary sources. There are a number of wild red-fleshed apples, and intense breeding has created red-fleshed apple varieties, because of the increasing interest in developing commercial red-fleshed apple varieties. Extensive crossbreeding programs involving good flavored, white-fleshed apples, have managed to improve the poor taste of the wild red-fleshed apple, producing a number of good tasting red-fleshed apples (Bars-Cortina et al., 2017). Recently, the discovery that the red flesh color in apple is under the control of the *MdMYB10* gene allowed the development of an alternative approach to produce a red-fleshed apple variety which could be compared to an isogenic, white-fleshed variety, by direct integration of the dominant *MdMYB10* allele into the “Royal Gala” line (Espley et al., 2009, 2013). Sensory and volatile profile analysis of these apples revealed no differences in flavor and aroma between *MdMYB10*-modified apple and the near-isogenic Royal Gala apple (Espley et al., 2013). However, consumption of red-fleshed *MdMYB10*-modified apple affected inflammatory pathway and gut microbiota in mice. After 7days of diet supplemented with *MdMYB10*-modified apple, expression of a group of cytokine genes linked to inflammation (*Interleukin-2 receptor B*, *Il2rb*; *CC motif chemokine receptor 2 and 10*, *Ccr2* and *Ccr10*; *C-X-C motif chemokine ligand 10*, *Cxcl10*) was decreased by twofold compared to mice fed with a diet supplemented with non-transformed Royal Gala apples. After 21days, mice fed with *MdMYB10*-modified apple showed a tenfold decrease in prostaglandin E2 (PGE2) and a non-significant decrease in leukotriene B4 (LTB4) plasma levels, compared with mice fed with Royal Gala apple. PGE2 and LTB4 are both inflammatory mediators derived from the arachidonic acid metabolism: the first, synthesized by cyclooxygenase 1 and 2 (COX-1, COX-2), is involved in the cardiovascular event associated with inflammation, while the second, a lipoxygenase product, is involved in the chemotaxis of leukocytes. Moreover, the gut bacterial flora changed in relation with the diet, and mice fed with *MdMYB10*-modified apple showed a significant decrease in *Lactobacillus* spp., whereas mice fed with Royal Gala apple experienced an increase in *Bifidobacterium* spp. (Espley et al., 2014). A similar study was conducted on 25 healthy adults in a randomized cross-over controlled trial using naturally bred red-fleshed apples or white-fleshed control apples. The analyses of fecal microbiota and of gene expression in peripheral blood mononuclear cells (PBMC, which consist of peripheral blood cells having a round nucleus, like lymphocytes and monocytes) have shown minimal differences between the two groups, but genes regulated by red-fleshed apples were immunoglobulin-related, suggesting a potential role in modulating the immune function (Barnett et al., 2021). Finally, both red- and white-fleshed apples seemed to have beneficial effects in hypercholesterolemic rats (Yuste et al., 2021) or in model rats with adenocarcinoma (Bars-Cortina et al., 2020). The benefits of apple and anthocyanin consumption are well established (Knekt et al., 2002; Boyer and Liu, 2004; Cassidy et al., 2015, 2016; Hyun and Jang, 2016), and apples with high anthocyanin contents could offer effective functional foods, to reduce the incidence of chronic diseases when part of a normal diet.

## Concluding Remarks

A considerable body of research has been devoted to identify the MYB-bHLH-WD40 transcription factors involved in the MBW complex activating the anthocyanin biosynthesis in many crop species, with the final aim of improving the anthocyanin content of plant-derived foods by means of conventional breeding or by metabolic engineering. Additional regulators of the anthocyanin pathway have been identified recently, acting as repressors disrupting the MBW complex or activators stabilizing the MBW complex. In addition, new levels of regulation have been described, in which the activity of repressors and activators is controlled by post-translational regulation. Anthocyanin production is also under epigenetic and post-transcriptional regulation (histone acetylation and miRNAs, respectively).

Despite research on positive and negative regulators has been extensive in the past decade, there are some gaps that could be filled. In some species, MBW complexes activating anthocyanins and related negative regulators still need to be identified. Are the negative regulatory systems identified in Arabidopsis, apple, and peach (e.g., MYBL2, SPL, JAZ, LBD, ANAC032, and AtSMX6) conserved in other species? Is TCP3 and its role as enhancer of MBW function and passive repressor of MYBL2 also conserved? Can these systems be exploited to enhance anthocyanin content in edible organs? How are R2R3-/R3-MYB repressors regulated in edible organs of fruits and vegetables and how can we modulate their expression in order to enhance anthocyanins, while avoiding their excessive undesirable accumulation? Further studies are required to understand the role of DNA methylation and histone modifications in anthocyanin repression both in model and crop species and eventually verified as a new possible approach to enhance organ-specific anthocyanin biosynthesis. Can miRNAs (e.g., *miR828*, and *miR858*) be silenced in edible organs to enhance anthocyanin accumulation? Are the lncRNAs newly identified in carrot conserved in other species? Can they be employed to enhance anthocyanin accumulation?

Advancing our knowledge of anthocyanin biosynthesis will allow the development of new biotechnological tools for the generation of value-added plants with increased anthocyanin content, which help consumers to achieve the desired amount of anthocyanins in their daily diet. Enrichment of foods in anthocyanins will also be particularly useful for the comparison of the nutritional properties of these bioactives from different food sources. Furthermore, the precise identification of biosynthetic genes encoding decorating enzymes in a specific plant food and a better understanding of their regulation will help in designing plant foods enriched in specific types of glycosylated/acylated anthocyanins. This may contribute to define which is the bioavailability and contribution to health-promoting properties of the single anthocyanin species present in a plant food, of their combination and eventually of the specific decorating groups in a food context.

## Author Contributions

All authors contributed equally to the manuscript, and read and approved the final manuscript. KP and CT contributed to conceptualization. FC, AM, MT, KP, and CT contributed to writing, reviewing, and editing.

## Funding

Financial support was provided by Fondazione Umberto Veronesi (Grant 2014 to KP; Grant Fellowships to FC and AM), EU FP7 project ATHENA (FP7-KBBE-2009-3/245121) to CT and the project MIND FoodS HUB (Milano Innovation District Food System Hub): Innovative concept for the eco-intensification of agricultural production and for the promotion of dietary patterns for human health and longevity through the creation in MIND of a digital Food System Hub, cofunded by POR FESR 2014-2020_BANDO Call HUB Ricerca e Innovazione, Regione Lombardia to KP and CT.

## Conflict of Interest

The authors declare that the research was conducted in the absence of any commercial or financial relationships that could be construed as a potential conflict of interest.

## Publisher’s Note

All claims expressed in this article are solely those of the authors and do not necessarily represent those of their affiliated organizations, or those of the publisher, the editors and the reviewers. Any product that may be evaluated in this article, or claim that may be made by its manufacturer, is not guaranteed or endorsed by the publisher.
